# Tailoring the Structure and Physico-Chemical Features of Cellulose-Based Hydrogels Using Multi-Epoxy Crosslinking Agents

**DOI:** 10.3390/gels10080523

**Published:** 2024-08-09

**Authors:** Raluca Nicu, Gabriela Lisa, Raluca Nicoleta Darie-Nita, Mihaela Iuliana Avadanei, Alexandra Bargan, Daniela Rusu, Diana Elena Ciolacu

**Affiliations:** 1 Department of Natural Polymers, Bioactive and Biocompatible Materials, “Petru Poni” Institute of Macromolecular Chemistry, 700487 Iasi, Romania; 2 Department of Chemical Engineering, Faculty of Chemical Engineering and Environmental Protection “Cristofor Simionescu”, “Gheorghe Asachi” Technical University, 700050 Iasi, Romania; gabriela.lisa@academic.tuiasi.ro; 3 Department of Physical Chemistry of Polymers, “Petru Poni” Institute of Macromolecular Chemistry, 700487 Iasi, Romania; darier@icmpp.ro (R.N.D.-N.); mavadanei@icmpp.ro (M.I.A.); 4 Department of Inorganic Polymers, “Petru Poni” Institute of Macromolecular Chemistry, 700487 Iasi, Romania; anistor@icmpp.ro; 5 Department of Physics of Polymers and Polymeric Materials, “Petru Poni” Institute of Macromolecular Chemistry, 700487 Iasi, Romania; rusu.daniela@icmpp.ro

**Keywords:** cellulose, epoxy-based crosslinker, hydrogels, ether bonds, swelling, rheological behavior, thermal degradation

## Abstract

Hydrogel features can be designed and optimized using different crosslinking agents to meet specific requirements. In this regard, the present work investigates the physico-chemical features of cellulose-based hydrogels, designed by using different epoxy crosslinkers from the same glycidyl family, namely epichlorohydrin (ECH), 1,4-butanediol diglycidyl ether (BDDE), and trimethylolpropane triglycidyl ether (TMPTGE). The effect of the crosslinker’s structure (from simple to branched) and functionality (mono-, bi- and tri-epoxy groups) on the hydrogels’ features was studied. The performances of the hydrogels were investigated through the gel fraction, as well as by ATR-FTIR, DVS, SEM, DSC, and TG analyses. Also, the swelling and rheological behaviors of the hydrogels were examined. The advantages and limitations of each approach were discussed and a strong correlation between the crosslinker structure and the hydrogel properties was established. The formation of new ether bonds was evidenced by ATR-FTIR spectroscopy. It was emphasized that the pore size is directly influenced by the crosslinker type, namely, it decreases with the increasing number of epoxy groups from the crosslinker molecule, i.e., from 46 ± 11.1 µm (hydrogel CE, with ECH) to 12.3 ± 2.5 µm (hydrogel CB, with BDDE) and 6.7 ± 1.5 µm (hydrogel CT, with TMPTGE). The rheological behavior is consistent with the swelling data and hydrogel morphology, such as CE with the highest Q_max_ and the largest pore size being relatively more elastic than CB and CT. Instead, the denser matrices obtained by using crosslinkers with more complex structures have better thermal stability. The experimental results highlight the possibility of using a specific crosslinking agent, with a defined structure and functionality, in order to establish the main characteristics of hydrogels and, implicitly, to design them for a certain field of application.

## 1. Introduction

Cellulose, with many benefits including its abundance and modest cost, along with biodegradability, high functionality, and unique morphology, remains a natural polymer of great interest and is mainly used in the development of new materials for the most varied and interesting applications [1,2]. These new materials include cellulose-based hydrogels, which are sometimes preferred over other hydrogels due to being highly biocompatible, easily affordable, and designable with fascinating structures and properties on a large scale and at a relatively low cost [3]. The presence of three hydroxy groups on each β-D-anhydroglucopyranose unit (AGU) of cellulose contributes to the formation of intra- and intermolecular hydrogen bonds, which plays a pivotal role in the design of cellulose-based materials. These OH groups can also function as reactive sites to form a variety of covalent linkages, with crosslinking being a feasible option [4].

The crosslinking process is of significant importance in designing permanent three-dimensional (3D) networks established within hydrogels and improving their physical and mechanical properties [5]. Depending on the type of crosslinking mechanism, two main types of hydrogels can be defined, namely physically and chemically crosslinked hydrogels. Physically crosslinked hydrogels are the result of the network involving weak and reversible interactions (i.e., ionic or electrostatic interactions, hydrogen bonds, van der Waals forces, and hydrophobic interactions) that occur between polymer chains and determine molecular entanglements or self-assembly [4]. One of the main advantages of physically crosslinked hydrogels involves the fact that it is not necessary to use chemical agents, possibly toxic, for designing 3D networks. However, physical networks have limited stability, because of the possibility of the established linkages to break relatively easily under stress in aqueous environments, thus restricting their possible applications [5]. This deficiency can be avoided by using chemical crosslinking agents in the preparation of hydrogels, thereby obtaining chemically crosslinked hydrogels with improved properties (i.e., material architecture, porosity, swelling degree, etc.), especially with respect to the control of structural stability and their mechanical properties [3]. Therefore, the choice of the chemical crosslinking agent is extremely important in controlling the physico-chemical characteristics of hydrogels [6,7,8,9].

Various types of chemical crosslinkers can be used, depending both on the biopolymer type and the extent of the properties’ improvement. In the design of cellulose-based hydrogels, a variety of crosslinking agents can be used, such as epichlorohydrin [1,10], aldehyde-based agents [11], adipic anhydride [12], or multifunctional carboxylic acids [13]. However, epichlorohydrin (ECH), a short epoxy molecule, also known as glycidyl chloride, is one of the most commonly used crosslinking agents. The crosslinking reaction occurs between the epoxy group of ECH and the hydroxyl group of cellulose (at any position) via an ether bond. This reaction takes place under alkaline conditions, using 6–9% NaOH solution, a commercially available low-cost and low-pollution solvent [14], without using a catalyst. The only co-products are NaCl and possible unreacted ECH, which can be easily washed out from the hydrogel once the reaction is complete. Moreover, even though ECH is considered to be toxic, it allows for obtaining materials free from crosslinker residues due to the high efficiency of the washing process [7]. All these facts lead to biocompatible cellulose-based hydrogels, supported by various studies regarding their applications, such as wound dressings in the biomedical field [15,16], as eco-friendly hydrogels with controlled release of fertilizer [17], as sustainable and superabsorbent hydrogels for removing contaminants from water [1], and so on.

The glycidyl ether family includes multi-epoxy compounds with a different number of epoxy groups in their structure (bi-, tri-, or multi-epoxy groups) and with different lengths of aliphatic, linear, or branched chains. The epoxy compounds are preferred when it comes to materials for medical applications due to the fact that they are considered less toxic compared with other crosslinking agents based on the ether bond, such as dialdehydes [18] or divinyl sulfone [19]. 1,4-Butanediol diglycidyl ether (BDDE) is a compound with bi-epoxy functional groups present at the two ends of its molecule, which promotes stable ether linkages with the OH groups of cellulose in alkaline environments [20]. Although unreacted BDDE should be considered as slightly to moderately toxic, the residual BDDE might undergo hydrolysis, leading to diol-ether, which has been proved to be non-toxic and to have limited safety risks [18,19]. For instance, due to its low sensitization and good biocompatibility, BDDE is widely used in the crosslinking of hyaluronic acid hydrogels in order to produce intradermal fillers and implants, and its clinical safety has been confirmed with a residual content of less than 1 ppm [20,21]. In another work, BDDE was used to crosslink hyaluronic acid and bacterial cellulose to obtain wound dressings with improved surfaces and mechanical properties, and thermal resistance [22]. Also, studies undertaken by Venzhik et al. [23] showed that the gels obtained by crosslinking carboxymethylcellulose with BDDE have reduced cytotoxicity, being proposed as materials suitable for medical use. Trimethylolpropane triglycidyl ether (TMPTGE) is a Y-shaped three-arm epoxy crosslinker, with an epoxy group at the end of each arm. High branching facilitates increased reactivity and crosslinking density. Thus, it is expected by its use to obtain materials with high bonding strength and water resistance [24]. For instance, TMPTGE was used as a crosslinking agent to prepare cellulose-based adhesives with great performance. The covalent bonds established between chains form a dense crosslinking network, which reduces the invasion of water molecules, so that the obtained cellulose-based materials show exceptional lap shear strength and water resistance [25].

Considering the high applicative potential of the cellulose-based hydrogels, as well as the constant search for developing new materials with tuned properties for various applications, we intended to design hydrogels starting from cellulose and three different crosslinking agents, namely epichlorohydrin (ECH), 1,4-butanediol diglycidyl ether (BDDE), and trimethylolpropane triglycidyl ether (TMPTGE), respectively. The crosslinking agents that were chosen for this study present different structures in terms of the number of epoxy groups contained in the compound, as well as in the branching of the structure, from simple to complex.

The aim of the study was to establish the correlation between the crosslinker structure/functionality and the physico-chemical features of their corresponding cellulose-based hydrogels, and to highlight the advantages and limitations of each approach. To the best of our knowledge, no comparative study regarding the use of three different crosslinking agents (ECH, BDDE, and TMPTGE) for the chemical crosslinking of cellulose has been reported in the literature to date. The designed hydrogels were investigated using several characterization methods, such as the gel fraction (GF) and swelling studies, dynamic vapor sorption (DVS), scanning electron microscopy (SEM), attenuated total reflectance Fourier transform infrared (ATR-FTIR) spectroscopy, oscillatory rheology, and thermogravimetric (TG) analysis, in order to cover a wide range of the features and to provide valuable information on the structure–property relationship for the obtained cellulose-based hydrogels.

## 2. Results and Discussion

There has been a continuous search for crosslinking agents that would allow for obtaining hydrogels with improved mechanical properties, good thermal stability, and adequate porosity while maintaining their high adsorption performance.

In this regard, the current study aims to investigate the features of three chemically crosslinked cellulose-based hydrogels, prepared by using three crosslinkers from the same glycidyl family: glycidyl chloride (ECH), 1,4-butanediol diglycidyl ether (BDDE), and trimethylolpropane triglycidyl ether (TMPTGE). The hydrogels obtained in this work were noted according to the type of crosslinker used, i.e., CE, CB, and CT. The evaluation of the morphology, physico-chemical properties, and rheological and thermal behaviors was performed by comparing the properties of the chemically crosslinked hydrogels both with each other and with those of the physically crosslinked cellulose-based hydrogel (C).

### 2.1. Interaction between Functional Groups of Cellulose and Epoxy Crosslinkers

Cellulose consists of repeating anhydroglucose units (AGUs), covalently linked between C4 and C1. The epoxy crosslinkers target the OH groups on the AGU, mainly at C2, C6, and C3 [26,27]. In this context, the crosslinking process is determined by the synergy between two processes: on the one hand, the chemical crosslinking processes, which involve the formation of ether linkages –C–O–C– between OH groups of the cellulose and those of the crosslinker by opening the oxirane ring; and on the other hand, the physical crosslinking process between the OH groups of the cellulose chains in close proximity to each other, with the formation of hydrogen bonds [16].

Under alkaline conditions (pH > 7), most ring structures of epoxy groups either open and covalently link to the most accessible hydroxyl groups in the polymer backbone of cellulose, forming stable ether bonds, or hydrolyze into an alcohol [19]. In addition, it has been demonstrated that a very small portion of the epoxy groups of the crosslinker, on the order of ppm, remain “un-opened” in a strongly alkaline environment [20].

Table 1 shows the structure of the three crosslinking agents used, together with the schematic representation of the most likely chemical crosslinking bond formations between the OH groups of the cellulose side chains and the epoxy groups of the crosslinking agent. As can be seen, the crosslinker can be present in different states: (i) fully reacted, where its molecule is involved in ether bonds with cellulose hydroxyl groups at all ends; (ii) pendant crosslinker, with a molecule that has reacted on one end only; (iii) deactivated or hydrolyzed crosslinker, in which case all the epoxy groups are converted into OH groups; and (iv) a residual crosslinker that has not reacted with cellulose or water (only trace amounts) [19]. These hypotheses are also supported by Ruhr and co-workers [28], in a study on the crosslinking of cellulose with a bifunctional epoxy group crosslinker, where it was stated that only some of the BDDE molecules reacted with the cellulose chains at both ends, establishing chemical crosslinking bonds, while the rest of the BDDE molecules reacted only at one end, and the other end either contained an unreacted epoxy group or was hydrolyzed during the process.

As expected, the more complex the structure of the crosslinker, the more complex the structure of the crosslinked network. In the case of ECH (Table 1a), which is a mono-epoxide molecule and the smallest among the three agents used, the crosslinking reaction is relatively simple regarding the formation of new ether bonds between the available OH groups on the cellulose chains and those formed by the opening of the oxirane ring. Its positioning and binding possibilities are relatively limited. However, with the increase in the number of functional epoxy groups in the structure of the crosslinker, the length of the aliphatic chain, as well as the degree of branching, the crosslinking reaction becomes more complex, increasing the orientation and positioning possibilities of ether bonds. For instance, the presence of the three epoxy groups at each end of the branched chains of TMPTGE (Table 1c) could allow for the formation of a much denser crosslinked network compared with ECH, having a positive effect on the mechanical stability and thermal properties of the hydrogel [25].

### 2.2. ATR-FTIR Investigations

ATR-FTIR spectroscopy was used as a reliable tool for the general characterization of the crosslinked hydrogels, because it highlights the spectral signatures of functional groups or newly formed bonds through the crosslinking reaction. In this regard, ATR-FTIR was used to establish the realization of the crosslinking reaction and the influence of each crosslinking agent’s structure on the cellulose-based hydrogels’ network formation.

**Figure 1:**
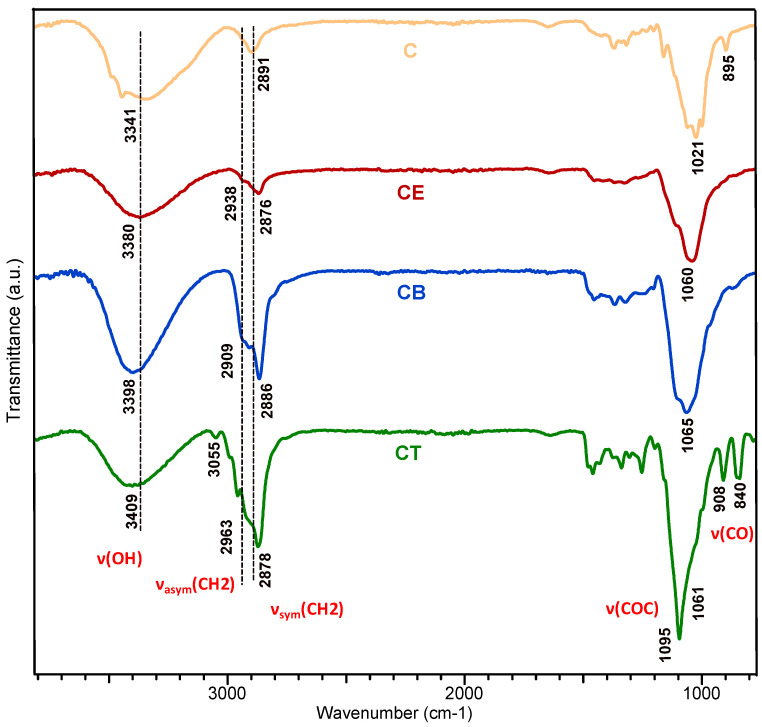
ATR-FTIR spectra of the cellulose-based hydrogels: physically crosslinked hydrogel (C); chemically crosslinked hydrogels with epichlorohydrin (CE), with 1,4-butanediol diglycidyl ether (CB), and with trimethylolpropane triglycidyl ether (CT).

ATR-FTIR spectra of the physically crosslinked hydrogel (C) and of the three chemically crosslinked hydrogels (CE, CB, and CT, respectively) are shown in Figure 1. All the spectra show the main characteristic bands, such as a broad band attributed to OH stretching vibrations, which are sensitive to inter- and intramolecular interactions (3380–3409 cm^−1^) [29,30], a C–H stretching band (2800–2960 cm^−1^) [1], O–H and C–H bending (1400–1300 cm^−1^) [31], C–O–H, C–O–C asymmetric stretching (1000–1200 cm^−1^) [18], and the absorption at 895 cm^−1^, which is characteristic of β-glucan in cellulose [32]. These bands show differences in terms of their intensity and width as a result of the use of the crosslinkers with different epoxy groups (mono-, bi-, and tri-) in the preparation of the hydrogels.

Indeed, the bands from 3200 to 3400 cm^−1^ of the chemically crosslinked hydrogels (CE, CB, and CT) recorded changes in comparison with the physically crosslinked hydrogel (C). More precisely, the band decreases along with the occurrence of a slight shift to the left (i.e., from 3341 cm^−1^ for hydrogel C to 3380 cm^−1^ for CE, 3398 cm^−1^ for CB, or 3409 cm^−1^ for CT, respectively), which might be attributed to the predominant involvement of OH groups in the formation of new ether bonds with the epoxy groups of the crosslinkers [18,33]. In the crosslinking process and, implicitly, in the achievement of the crosslinked network, it is supposed that the OH groups are mainly connected through the opening of the epoxy ring. As the epoxy ring is opened and reacts with OH groups corresponding to the cellulosic chain, the number of free OH groups shows a downward trend [25]. This fact can explain the slight decrease in the intensity of the band at 3341 cm^−1^ for hydrogel CT, in which most of the OH groups are involved in the crosslinking bonds. However, for hydrogel CB, this band shows an increase, being susceptible to an increase in OH groups through the opening of oxirane rings in alkaline medium, without the participation of all OH groups in the formation of ether bonds.

Moreover, significant intensity changes can be observed for the bands in the region from 2800 to 2900 cm^−1^, associated with the contribution of aliphatic moieties (–CH_2_, methylene symmetric, and asymmetric stretching, respectively) [34], especially for hydrogels CB and CT. In these cases, the crosslinkers BDDE and TMPTGE are used, in whose structure long aliphatic chains predominate, compared to the small molecule of ECH, confirming the incorporation of the crosslinker into the cellulose network. The increase is more obvious when TMPTGE is used as a crosslinker, with longer and more branched aliphatic chains.

The absorption bands that confirm the achievement of chemical crosslinking by the formation of new ether (C–O–C) bonds are the bands in the 1050–1200 cm^−1^ region [10,17], more exactlyat 1060 cm^−1^ for hydrogel CE, 1065 cm^−1^ for hydrogel CB, and 1061 cm^−1^, as a shoulder, for hydrogel CT, respectively. However, for hydrogel CT, two small peaks can be observed at 908 cm^−1^ and 840 cm^−1^, characteristic of the stretching vibration of C–O in glycidyl esters [35], indicating that a small portion of the epoxy ring at the end of TMPEG did not open. This is also suggested by the band at 3055 cm^−1^, characteristic of the –CH_2_ stretching vibration of terminal epoxides, which indicates the ring opening did not occur for all the epoxy groups [33].

### 2.3. Morphological Analysis of Hydrogels

The cross-sectional morphologies of the cellulose-based hydrogels were analyzed by SEM in order to obtain relevant information about the pore geometry, apparent pore size, and heterogeneity of the overall hydrogel’s network. It is important to investigate the morphology of the hydrogels before other properties because the pore structure greatly influences essential features, such as interactions with water molecules in the liquid state (i.e., swelling degree) and in vapor state (i.e., adsorption/desorption isotherm and hysteresis), and the rheological behavior.

**Figure 2:**
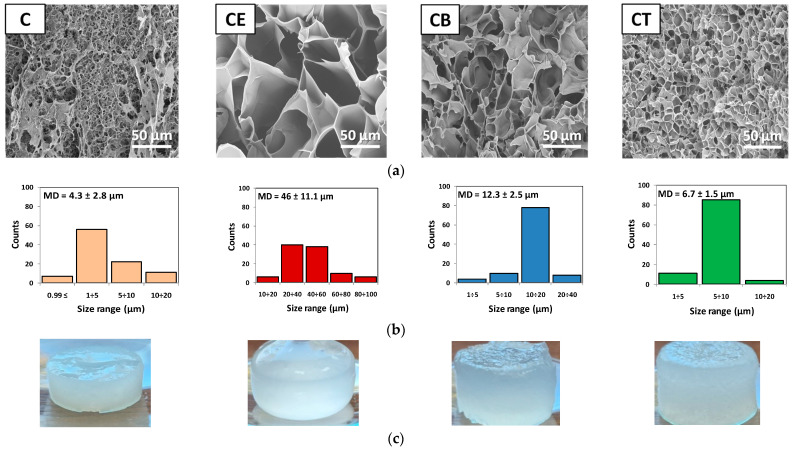
(**a**) Cross-sectional micrographs (mag 500×), (**b**) pore size distribution histogram (MD—mean pore diameter), and (**c**) photographs of never-dried hydrogels.

According to cross-sectional SEM images presented in Figure 2a, the obtained 3D networks have totally different morphologies from each other. Thus, the physically crosslinked hydrogel (C) exhibits an irregular pore structure formed mainly by the entanglement and self-association of the cellulose chains, while the chemically crosslinked hydrogels (CE, CB, and CT) have different 3D network morphologies and porosities depending on the type of crosslinker used, and implicitly, the established crosslinking bonds. As expected, by using crosslinkers with an increased number of epoxy groups, the pore size decreases and the matrix becomes denser, more compact, and even more uniform. Similar results reported by Luo and coworkers [36] showed that by crosslinking with BDDE, the obtained composites based on bacterial cellulose and hyaluronic acid were much denser and had smaller pores than those from unmodified bacterial cellulose. Moreover, from Figure 2c, it can be seen that the presence of the crosslinker in the system determined the destruction of the self-association and packing of the cellulose chains [37], leading to obtaining more swollen and almost translucent hydrogels (see hydrogel CE), with a more expanded structure.

It was observed that the physically crosslinked hydrogel (C) had a discontinuous porous structure, while the most uniform and compact network, with smaller and regular pore sizes, was obtained for hydrogel CT, where the mean pore diameter (MD) was 6.7 ± 1.5 µm (Figure 2b). This result can be attributed to the TMPTGE crosslinker, with a Y-shaped structure and three arms that permits the formation of new ether bonds with three different cellulose chains at the same time, leading to a more compact structure than that obtained with ECH, for example. Even if ECH is the smallest molecule, this leads to obtaining 3D networks with large, well-defined pores (MD = 46 ± 11.1 µm) with an elongated and irregular shape that are slightly interconnected with visibly thicker walls. For hydrogel CB obtained with the bi-epoxy crosslinker (BDDE), the pores are not as small and uniform as in the case of the CT matrix (MD = 12.3 ± 2.5 µm), but their interconnectivity is much higher than those of the CE and CT hydrogels, which plays a crucial role in the diffusion of the water molecules.

Thus, it can be concluded that the differences between the structure/functionality of the three crosslinking agents are directly reflected both in the pore shape and in the uniformity of pore distribution. With the increase in the number of epoxy groups, pores with an aspect ratio (i.e., the ratio of minor to major pore length) close to unity are obtained, predominantly spherical for hydrogels CB and CT, and the porous structure tends towards high uniformity (i.e., the majority of the pore size, around 80%, is in the size range of 5–10 µm for CT and 10–20 µm for CB, respectively) (Figure 2b). Instead, hydrogel CE has pores with an aspect ratio less than unity with a predominantly elongated, cylindrical shape, and the hydrogel structure is non-uniform (i.e., the pore size covers a much wider range and the standard deviation has the highest value ±11.1 µm).

### 2.4. Gel Fraction Measurements

Generally, the formation of a 3D network of hydrogels occurs by chemical or physical crosslinking. To quantify the amount of crosslinking bonds established in the 3D network, the gel fraction (GF) method is used, which is the most effective way to evaluate the degree of crosslinking. Consequently, a hydrogel with a higher gel fraction indicates a higher crosslinking degree.

Table 2 presents the gel fraction (GF, %) and the amount of crosslinker (AC) consumed per unit of anhydroglucose unit (AGU), namely mole_AC_/mole_AGU_ for the obtained hydrogels. It can be observed that the same amount of crosslinker and cellulose (M_C_) leads to different weights of dry hydrogel (M_H_) and contributes in a different way to the formation of the 3D network, expressed by the M_H_/M_C_ ratio. For hydrogel C, only 74% from the initial amount of cellulose is converted into a physically crosslinked hydrogel. This result can be explained by the loss of some proportion of the hydrogel through repeated washings with warm distilled water, due to the high susceptibility of intermolecular hydrogen bonds to water. However, after the drying process, the entanglement and self-association of the cellulose chains that occur in hydrogel C become stronger, so that the resultant hydrogel is stiffer, which explains its high GF of 96.3%.

Regarding the chemically crosslinked hydrogels (CE, CB, and CT), the situation is different from that of the physically crosslinked hydrogel. Although the same amount of crosslinker is used for all hydrogels, it is consumed differently depending on the number of epoxy groups located at the ends of the aliphatic, linear, or branched chains. Among the three chemically crosslinked hydrogels, the highest GF is obtained for hydrogel CE, namely 97.3%. This fact demonstrates the high capacity of ECH to form ether bonds with the OH groups of the cellulose chains (only 0.876 mole per mole of AGU is consumed), which allows for the formation of a stronger crosslinked network, with the ability to maintain its physical integrity, without dissolving in an aqueous medium [27]. A GF of over 94% is also obtained for hydrogel CB, which is prepared by using BDDE, with a consumption of 1.843 moles of AC per mole of AGU, double that of ECH. However, the situation is different for hydrogel CT, which has a GF of only 82.3%. In this case, it is assumed that due to the large size of the crosslinker, a “steric effect” is involved to a certain extent in the crosslinking process. Thus, it is possible that the three-arm branched crosslinker (TMPTGE) does not have access to all the OH groups of the cellulose and, implicitly, cannot establish many of the possible ether bonds, remaining partially reacted or even unreacted, and then being removed through the washing process. This hypothesis is also confirmed by the ATR-FTIR investigations (see Figure 1), where the spectrum of the CT hydrogel indicates the fact that a certain proportion of oxirane rings did not open in the alkaline environment, remaining unavailable for crosslinking.

### 2.5. Swelling Behavior and Swelling Kinetics

The swelling degree is another way to assess the effectiveness of hydrogel crosslinking [5]. Usually, the water content of a fully swollen hydrogel is directly proportional to the crosslinking density, but also to matrix porosity [3]. The swelling ability was measured by the conventional gravimetric method, and the values of the maximum swelling degree (Q_max_, %), corresponding to the never-dried hydrogels, and the swelling degree (Q_t_, %), determined for the hydrogels dried by lyophilization, are presented in Table 3.

The swelling degree depends both on the type of crosslinking (physical or chemical) and on the type of crosslinking agent (mono- or multi-epoxy, linear or branched). In terms of swelling ability, it is expected that chemically crosslinked hydrogels will present higher water absorption than physically crosslinked hydrogels, considering that by the crosslinking reaction, the compact chain packing of cellulose chains is disturbed, leading to the enhancement of chain mobility, and implicitly, of hydrophilicity [27]. However, this situation is valid only for CE, but not for hydrogels CB and CT, which have a lower swelling degree (Q_t_ = 580% and 470%, respectively) than the physically crosslinked hydrogel C (Q_t_ = 710%). These results can be attributed to the involvement of several factors, such as those related to the network characteristics: the structure and shape of the pores, the thickness of the pore walls, the uniformity of pore distribution, and even the drying process.

Moreover, there is a noticeable difference in terms of swelling degree between the never-dried hydrogels (Q_max_) and the hydrogels dried by lyophilization (Q_t_).

Hydrogel CE has the advantage of a structure with large pores, which explains the higher swelling degree recorded both in the never-dried state (Q_max_ = 1280%) and after drying (Q_t_ = 1030%). Moreover, the structure of hydrogel CE is characterized by pores with well-defined and thicker walls (see SEM, Figure 2a), so that most of it is preserved after drying, and implicitly, the ability of the hydrogel to absorb water is also maintained (CE loses only 19% of its swelling capacity).

The never-dried C, CB, and CT hydrogels show almost similar swelling degrees, which fall within the range of Q_max_ = 940–970%. However, after drying by lyophilization, the physically crosslinked hydrogel, C, records a loss of swelling capacity of approx. 26%, the chemically crosslinked hydrogel CB of approx. 38%, and the most affected is CT, whose swelling capacity decreases by 50% (from 970% to 470%). An explanation can be the fact that the “never-dried” state of the hydrogels is characterized by a relaxed network and an expanded state. However, through the gradual removal of water during the lyophilization stage, a rearrangement of the network occurs (cornification phenomenon) through the formation of new hydrogen bonds between the polymer chains, and even the collapse of certain pores with very thin walls, which leads to denser networks with lower swelling capacity [6,17].

Regarding the pronounced decrease in the swelling degree of chemically crosslinked hydrogels (CB and CT), a major influence is exerted by the presence in their structure of longer aliphatic chains due to the use of crosslinking agents, such as BDDE and TMPTGE, respectively. During the drying process, they allow for the establishment of a greater number of hydrogen bonds, and the more complex the structure of the crosslinker, the more noticeable the decrease in swelling degree (Q_t_ CE > Q_t_ CB > Q_t_ CT).

**Figure 3:**
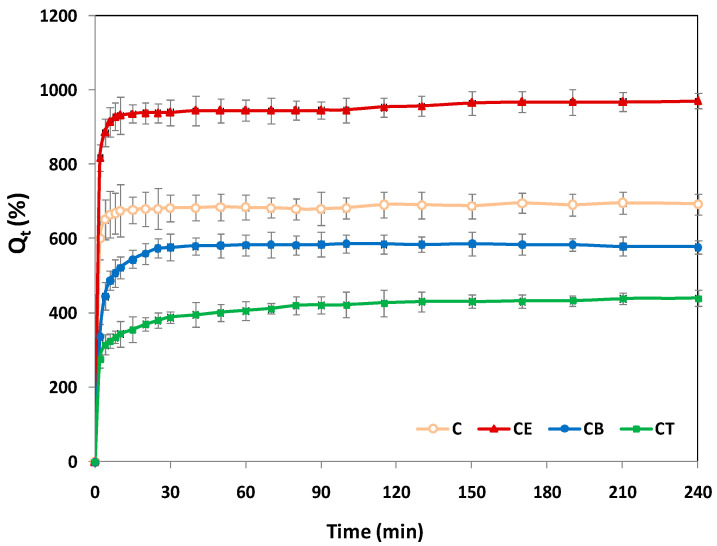
Dynamic swelling behavior of cellulose-based hydrogels: physically crosslinked hydrogel (C) and chemically crosslinked hydrogels with epichlorohydrin (CE), 1,4-butanediol diglycidyl ether (CB), and trimethylolpropane triglycidyl ether (CT).

Precious information regarding the phenomenon of water molecule absorption and the behavior of 3D networks in the water uptake process can be provided by studies of swelling kinetics, using empirical Equation (6) [38]. The dynamic swelling behavior of all four hydrogels is presented in Figure 3. The swelling capabilities of all the cellulose-based hydrogels increase with time, so that after a certain period, they reach a steady state, a plateau. As shown in Figure 3, the equilibrium swelling state for all the hydrogels is reached in approximately 30 min, and for the CB and CT hydrogels, reaching the plateau is much slower. For instance, in the first 5 min, almost 80% of the total amount of water is absorbed for C and CE, while in the case of CB and CT, this percentage is much lower, below 50%. This phenomenon can be seen as an advantage of the structures crosslinked with BDDE and TMPTGE, respectively, which present a slower swelling process, controlled more by the diffusion process than by chain relaxation.

The kinetic parameters, which are indicative of the water transport mechanism (i.e., the swelling constant, k_sw_, and the swelling diffusional exponent, n_sw_) can be found in Table 3 and are in agreement with the results obtained and presented above. The perfect Fickian diffusion process of the water molecules through the porous structure of the hydrogels is characterized by a diffusion coefficient of n_sw_ = 0.45 (i.e., for cylindrical hydrogels) and the relaxation of polymer chains in the swelling process equals the diffusion of water molecules through the porous structure. In the case of the physically crosslinked hydrogel, C, the diffusion coefficient has the lowest value (n_sw_ = 0.038), which indicates that the water transport mechanism is based more on the relaxation phenomenon of the polymer chains and less on the diffusion phenomenon. On the other hand, for chemically crosslinked hydrogels (CE, CB, and CT), in the structure of which there are relatively stable ether chemical bonds, the water diffusion phenomenon prevails to the detriment of the relaxation of the polymer chains, illustrated by the approximately three-fold increase in the diffusion coefficient values (n_sw_ = 0.094–0.111). For all studied hydrogels, the correlation coefficient (R^2^) has values greater than 0.99 (between 0.992 and 0.997), which suggests an excellent fit between the experimental data and the chosen model.

### 2.6. Dynamic Vapor Sorption Measurements

The water adsorption/desorption behavior is important in assessing the porous structure of the materials, establishing their stability in environments with a specific relative humidity (RH), understanding how water molecules migrate within their structure, and determining how they gain or lose water [39]. Furthermore, studies on different types of sorption isotherms provide crucial information regarding the most appropriate mathematical model that the experimental data can be correlated with, and moreover, provide information on the interdependence of physical structure and macroscopic behavior [40]. In our particular case, DVS has been used to study the interaction of the hydrogels with water vapor, to evaluate surface properties, and to highlight the influence of each type of crosslinker on these properties.

**Figure 4:**
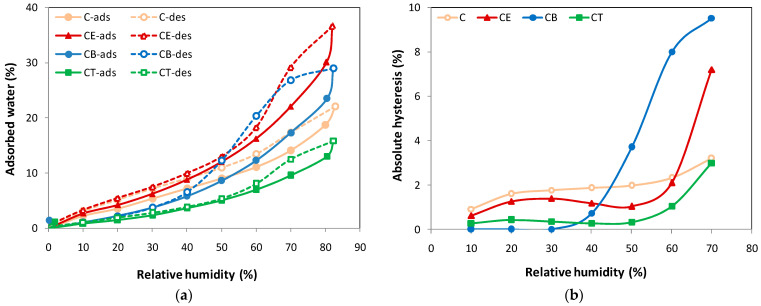
Dynamic vapor sorption data for cellulose-based hydrogels: (**a**) adsorption/desorption isotherms (adsorption—solid line; desorption—dashed line) and (**b**) absolute hysteresis vs. relative humidity.

The relation between the equilibrium moisture content and the relative humidity, at constant temperature, as well as the hysteresis phenomenon for cellulose-based hydrogels are presented in Figure 4a,b. The adsorption/desorption isotherms (Figure 4a) indicate the dependence between the water content of the hydrogels and the relative humidity (RH). For instance, each value of RH in the sorption isotherm corresponds to a certain value of water content, at a given constant temperature, for a specific type of hydrogel. According to the International Union of Pure and Applied Chemistry (IUPAC) classification, the curves shown in Figure 4a fit on a type V isotherm, characterized by reduced water vapor adsorption at low values of RH (0–10%), moderate adsorption at intermediate values of RH (20–50%), and a sharp increase in water adsorption for RH values higher than 50% [41].

As shown in Figure 4a, a very low water content (<5%) is recorded for all the hydrogels for a relative humidity of up to 30%, indicating the possibility of the formation of a monomolecular water layer on the surface of the solid (monolayer water adsorbed) [40]. It should be mentioned that with increasing relative humidity, up to 50%, the behavior of the hydrogels in the presence of water vapor is similar to that observed in the case of the swelling degree, where Q_t_ decreases according to the following series: Q_t_ CE > Q_t_ C > Q_t_ CB > Q_t_ CT. At this point, a water content of approximately 12% is obtained for CE, 9% for C, 8.6% for CB, and only 5.5% for CT hydrogels, respectively. Above a value of 50% RH, significant changes are recorded in the shape of the vapor adsorption isotherms corresponding to hydrogels and, implicitly, in the interaction between water vapor molecules and the solid material. A more special evolution can be observed for the CB hydrogel, which with a significant increase in RH, adsorbs double the amount of water vapor compared to the CT hydrogel. This fact is also found in the case of the swelling degree of the hydrogels, when Q_t_ is higher for CB than for CT. Finally, at approximately 85% RH, the maximum amount registered for adsorbed water vapor (W, %) is 36.6% for CE, 28.9% for CB, 22% for C, and only 15.8% for the CT hydrogel (Table 4).

Unlike the swelling process, where the results are due to a combination of factors (nature of the fibers, solvent properties, specific process conditions, etc.), the adsorption/desorption of water vapor, especially for RH > 50%, is overwhelmingly influenced by the pore shape and interconnectivity, namely whether the pores are cylindrical, spherical, open-ended, ink-bottle-shaped, and so on. Experiments show that the large hysteresis loops recorded at high RH (usually at values > 75%) arise either due to differences in condensation/evaporation occurring in “ink bottle” pores or due to differences in the meniscus during adsorption/desorption in cylindrical pores [42,43]. In our case, this can be seen in the isotherms corresponding to the CE and CB hydrogels, shown in Figure 4a. Also, there are notable differences among the four hydrogels, not only in the position of maximum hysteresis, but also in its magnitude (Figure 4b). The lower hysteresis observed for the C and CT hydrogels, at 55–75% RH, may be an indication of the weaker interactions of its active sites with water, and consequently, of the smaller structural changes due to polymer swelling. The highest magnitude of loop hysteresis is observed for the CB hydrogel. It should be mentioned that valuable information can be obtained from the shape of desorption branch, regarding the size and shape of the pores, as well as about the predominant mechanism of filling or emptying the pores. This is the case of “inkbottle” type pores, where wide pores have access to the external surface only through narrow necks. The wide pores remain filled during the desorption process, until vapor pressures are low enough to allow for their emptying through the narrow necks. If the neck diameter is not too small, then the network can empty even at higher relative pressures.

In our particular case, depending on the shape of the adsorption/desorption isotherms and the hysteresis loop size, we can assume that hydrogels C and CT have predominantly spherically shaped pores, hydrogel CE has predominantly cylindrically shaped pores, while hydrogel CB is much more special, containing a large amount of “ink bottle” type pores, which determine the appearance of the largest hysteresis loop. The presence of this type of pore in the porous structure of the CB hydrogel is also confirmed by the higher values obtained both for the specific surface area (approximately 375 m^2^/g) and monolayer (0.1068 g/g) (Table 4). This special structural characteristic of the CB hydrogel, although not characterized by a high degree of swelling, can still be seen as an advantage in applications that require high water retention over a long period of time, such as in (i) the field of agriculture, to maintain a certain humidity in the soil in order to ensure the optimal development of plants, or even in (ii) the biomedical field for wound healing, to make wound dressings that maintain a favorable environment for a long time.

### 2.7. Rheological Behavior

**Figure 5:**
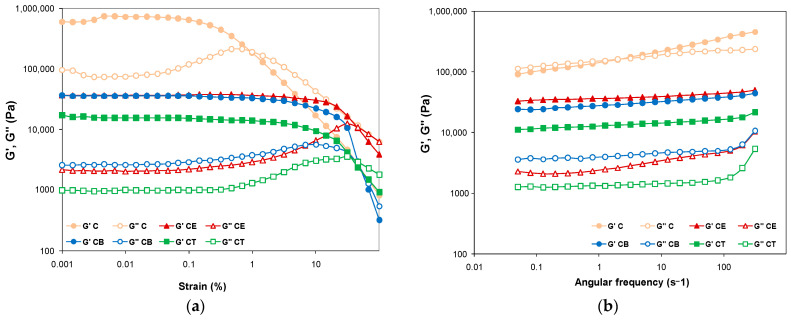
The dependence of dynamic moduli on strain and angular frequency: (**a**) amplitude sweep test results; (**b**) frequency sweep test results (G′—solid symbols; G″—open symbols).

Rheology is an effective tool to characterize the viscoelastic behavior of hydrogels. Rheological properties are evaluated in terms of the storage modulus (G′), which refers to the elastic component of the material, whereas the viscous component is given by the loss modulus (G″). More specifically, the elastic modulus (G′) describes the solid-like behavior of the gel, whereas the loss or viscous modulus (G″) defines its liquid-like behavior [44]. The first step in the evaluation of the dynamic viscoelastic properties of our developed hydrogels was to perform amplitude sweep tests. Different values of the storage and loss moduli were obtained for the four types of studied hydrogels, by varying the strain from 0.001 to 100%, as shown in Figure 5a, hinting at a clear dependence of the rheological behavior on the crosslinker structure and functionality [6]. As a general remark, the storage modulus of all the hydrogels dominates over the loss modulus up to the crossover point, suggesting that the elastic behavior of all the hydrogels prevails over their viscous behavior, demonstrating a typical gel-like behavior [45,46].

For instance, in the linear viscoelastic region (LVR), where the moduli are strain-independent, values of ~604 kPa (C), 36 kPa (CE, CB), and 17 kPa (CT) are obtained for G′. Although the physically crosslinked hydrogel (C) surprisingly presents the highest value for G′ and exhibits the most enhanced stiffness “at rest” of the network [10], the chemically crosslinked cellulose hydrogels can withstand higher deformation than hydrogel C. Similar results were also reported in previous studies, where the physical hydrogels exhibited a much higher storage modulus, being even 100–200 times greater than that of chemically crosslinked hydrogels. The explanation was based either on the formation of lumps by macromolecules [45] or on the strong development of inter- and intra-molecular hydrogen bonds by the entanglement and self-association of the cellulose chains after the freezing/thawing process, decreasing the free volume [47]. The storage moduli of all the evaluated hydrogels crosslinked with EPC, BDDE, and TMPTGE in LVR are in the range of 17–40 kPa, falling within the value range reported for other chemically crosslinked cellulose-based hydrogels [48,49].

The values of the dynamic moduli (G′ = G″) and strain (γ, %) at the crossover point can be determined from the graph of Figure 5a and are observable in Table 5. All three chemically crosslinked hydrogels (CE, CB, and CT) show the crossover point beyond ~40% strain, reflecting a much higher stability of these hydrogels [6] compared to the physically crosslinked one, where the crossover of G′ and G″ occurs at a very low strain value of less than 1%. Among the chemically crosslinked hydrogels, CT shows the lowest stability during strain deformation. A similar result was obtained by Su Ting and coworkers [24], where the linear crosslinker with two-arms (BDDE) led to a higher storage modulus and a more stable gel than the one with three-arms (TMPTGE).

Beyond the value of the crossover point, as can be seen in Figure 5a, the viscous regime dominates (G″ > G′) and all viscoelastic moduli become dependent on the strain amplitude, decreasing as a result of the structural breakdown of the hydrogel network under high deformation [45].

**Figure 6:**
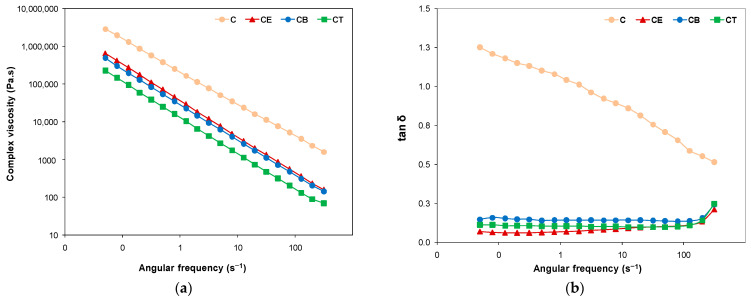
The rheological behavior of cellulose-based hydrogels: (**a**) complex viscosity and (**b**) loss factor dependence on the angular frequency.

The next step of the dynamic viscoelastic property evaluation was to perform the frequency sweep tests, the corresponding results being summarized in Figure 5b and Figure 6, as well as in Table 5. The physically and chemically crosslinked cellulose hydrogels exhibit G′ moduli greater than G″ moduli, indicating gel-like viscoelastic behavior in the tested frequency range. It is worth noting that, for sample C, both dynamic moduli slightly depend on the frequency in the investigated experimental domain, more obviously for the G’ at higher frequencies. The value of G′ and G″ at the crossover point is 162 kPa at the angular frequency of ω = 2.1 s^−1^, so this physically crosslinked cellulose hydrogel shows increased stiffness (Figure 5a). In the case of the CE, CT, and CB hydrogels, both dynamic moduli, and especially G′, are nearly independent of the angular frequency. Hence, it can be mentioned that the stability of these hydrogels’ network structure is not affected by the applied deformation, with the hydrogels displaying an excellent structured 3D network. Moreover, the lack of a crossover point of viscoelastic moduli suggests permanent chemical crosslinking [10].

The influence of the polymer network structure resulting from chemical crosslinking was revealed by the rheological results as the stiffness of CE, CT, and CB decreased relative to hydrogel C, as also observed by the values of G′ at 5 s^−1^ displayed in Table 5. We have chosen to evaluate the values of G′ and G″ at 5 s^−1^ for a better comparison of the moduli evolution in the middle of the tested frequency range. The decline in G′ values by crosslinking with EPC, BDDE, and TMPTGE shows a more elastic structure of the resulting materials, which is a highly desired feature for hydrogel applications, especially in the medical field. An observation could be that both G′ and G″ values reduce with the increase in the molecular weight of the crosslinkers used, thus leading to a decline in the entanglement degree, so that the polymer chains move more easily under shear stress [50].

The changes in the hydrogels’ network are also reflected in their complex viscosity dependence on angular frequency, lower values being registered for the flexible chemically crosslinked hydrogels compared with the stiffer hydrogel C (Figure 6a).

The relative contribution of the viscous components to rheological properties was evaluated through the loss factor (tan δ), which is defined as the ratio between viscous to elastic response (tan δ = G″/G′) and reflects the damping abilities of the hydrogel. The lower tan δ values for chemically crosslinked hydrogels indicate enhanced elasticity, because tan δ represents the ratio of dissipated to stored energy [48]. Figure 6b presents the loss tangent evolution vs. frequency, and Table 5 shows the values of this parameter at ω = 5 s^−1^. As can be seen in Figure 6b, there is a big difference between the physically crosslinked hydrogel and the three chemically crosslinked hydrogels, and that there is no noticeable difference in the loss tangent among the latter. Tan δ values of CE, CB, CT hydrogels are in the range of 0.06–0.3 and are almost constant over the studied frequencies for all the hydrogels, proving that the elastic properties are superior to the viscous properties in terms of the dynamic viscoelastic behavior [51]. Tan δ reflects the superposition of G′ with G″ (tan δ = 1) for hydrogel C, with values decreasing from 1.3 to about 0.5, denoting a gradual shift from a viscous liquid at low frequencies (G″ > G′, tan δ ~ 1) to a soft viscoelastic solid (tan δ ~ 0) at higher frequencies.

As a general conclusion following from these rheological tests, we could say that the viscoelastic properties evaluated by dynamic rheology are correlated with the changes in the structure of the crosslinked hydrogels, especially with respect to their degree of swelling and porosity.

### 2.8. Differential Scanning Calorimetry and Thermogravimetric Analyses

Generally, DSC is a technique that measures the heat capacity (heat flow) as a function of temperature, in a controlled atmosphere, and can provide information on thermally induced conformational transitions and phase transitions of polymeric materials, which can be associated with the absorption of heat (endothermic: evaporation of water, thermal decomposition of polymers, melting of solids, denaturation of proteins, etc.) or with the release of heat (exothermic: crystallization of polymers, aggregation of proteins, etc.) [13].

Hydrogels, by their nature, are hygroscopic materials that absorb water vapor from the environment until reaching an equilibrium moisture content, which may be present as adsorbed moisture on internal surfaces and as capillary condensed water in pores. This process is closely dependent on the porous structure of the network, but also on the OH groups on the surface of the polymer chains, which are available to bind water molecules [41]. The more OH groups there are, the more difficult it will be to remove water from the hydrogel structure with an increase in temperature (heating).

**Figure 7:**
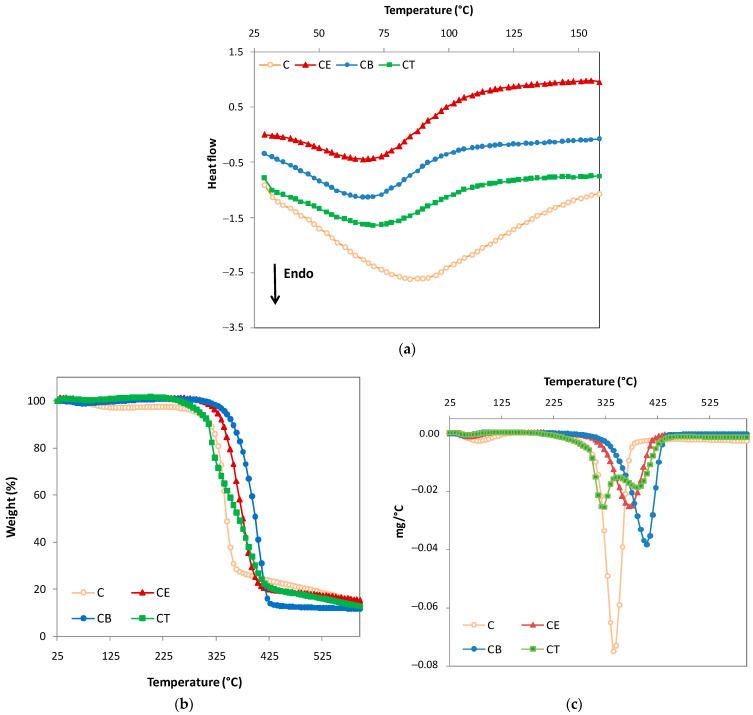
(**a**) DSC, (**b**) TG, and (**c**) DTG curves of cellulose-based hydrogels (physically crosslinked hydrogel—C; chemically crosslinked hydrogels with epichlorohydrin—CE; with 1,4-butanediol diglycidyl ether—CB; with trimethylolpropane triglycidyl ether—CT).

Figure 7a shows the DSC thermograms corresponding to the water evaporation process, which is highlighted by a single endothermic peak, with a maximum temperature (T_peak_) located between 70.6–84.6 °C, depending on each type of hydrogel in terms of whether it is physically or chemically crosslinked. As shown in Table 6, the endothermic peaks (T_peak_) appear at 84.6 °C for hydrogel C, 76.6 °C for CE, 70.6 °C for CB, and at 71.8 °C for CT. For hydrogel C, which presents the largest number of hydroxyl groups available for hydrogen bonds, a broad endothermic peak associated with water loss is recorded at the highest values of T_peak_.

These results are in agreement with the DVS data, where in the case of hydrogel C, by decreasing the RH from 40% to 10%, a slower water loss is observed, also suggested by the higher value of the absolute hysteresis in this RH range, compared to the other hydrogels (see Figure 4b). Through the chemical crosslinking process, the hydroxyl groups are more involved in the establishment of ether bonds, thus recording a decrease in the number of water binding sites, which is confirmed in the DSC thermograms of hydrogels CE, CB, and CT by a shift in the maximum temperature of the endothermic peak towards lower temperatures. Dehydration heat values (ΔH, J/g) show the same trend for the chemically crosslinked hydrogels compared to the physically crosslinked ones.

The thermal stability of the hydrogels is closely related to the structure of the network and their crosslinking density. TG analysis was chosen to investigate the thermal stability of the hydrogels, both physically and chemically crosslinked. This technique is a sensitive instrumental technique used to monitor mass changes as a function of temperature and to provide information on degradation processes and thermal stability. Figure 7b,c presents the thermograms (TG, Figure 7b) and derivative thermogravimetric curves (DTG, Figure 7c) of the studied hydrogels. Furthermore, all thermal decomposition parameters, including T_onset_, T_peak_, T_endset_, weight loss at the end of each thermal decomposition stage, and total residue, are presented in Table 7.

As a general observation, the thermal degradation of the cellulose-based hydrogels takes place in two main stages. An exception is highlighted for hydrogel CT, in which an “intermediate” stage corresponding to the “free” TMPTGE crosslinker appears, recorded for the peak with T_peak_ of 319 °C. This result can be explained by a partial consumption of the crosslinking agent during the crosslinking reaction and the fact that “free” TMPTGE could not be removed by repeated washings. This observation is confirmed by ATR-FTIR spectroscopy, where an absorption band characteristic of unopened oxirane rings from TMPTGE is identified in the CT hydrogel spectrum (see Figure 1).

The data obtained from the first stage of weight loss (Stage I, Table 7) are in agreement with those obtained from the DSC analysis, emphasizing the fact that the mass loss in this stage is due to the loss of absorbed moisture from the hydrogel’s network. In addition, these data confirm the relatively slower water loss process for the physically crosslinked hydrogel (C) compared to the chemically crosslinked ones.

In the second stage, which represents the thermal degradation stage, there is a substantial loss of mass between 83.2% for C and 90.1% for CB hydrogels. This is related to the cleavages of the ether bonds occurring between cellulose chains (for chemically crosslinked hydrogels CE, CB, and CT), to the breaking of glycosidic linkages of cellulose skeleton (also including the physically crosslinked hydrogel, C) [52], as well as to the complete degradation of the hydrogels, with the formation of gaseous molecules (such as CO, CO_2_, H_2_O, etc.), into a carbon-rich residue. The physically crosslinked hydrogel has the lowest thermal stability because it starts degrading at T_onset_ = 318 °C, while the CB hydrogel begins degrading at 345 °C, the highest temperature among the studied hydrogels. Moreover, CB has the highest T_peak_ at 403 °C, showing the highest thermal stability (CE, T_peak_ at 372 °C and CT, T_peak_ at 388 °C). However, the thermal behavior of all the three chemically crosslinked hydrogels is relatively similar, indicating that the structure and functionality of the crosslinker has a slight effect on their thermal stability. A notable difference appears only when comparing them with the physically crosslinked hydrogel, revealing a clear indication of the formation of a crosslinked network with a more thermally stable structure.

## 3. Conclusions

In this study, cellulose-based hydrogels were successfully designed, using physical or chemical crosslinking, in the presence of multi-epoxy crosslinking agents (mono-, bi-, and tri-epoxy). The two-step technique for the preparation of these hydrogels included (i) a dissolution step of cellulose in aqueous NaOH solution to trigger the cellulose gelation process in strong alkaline medium, followed by (ii) a crosslinking step at a relatively low temperature (40 °C) in order to obtain the chemically crosslinked hydrogels. The performances of the engineered hydrogels were systematically investigated through the gel fraction and swelling degree evaluation, the rheological behavior, and SEM, DVS, DSC, and TG analyses.

The obtained results suggest that by using different crosslinking agents such as ECH (mono-epoxy), BDDE (bi-linear epoxy), or TMPTGE (three-branched epoxy), chemically crosslinked hydrogels with relatively different characteristics can be obtained. Significant differences were observed in terms of network morphology and pore size distribution, as well as in gel fraction values, which together synergistically contributed to the design of all other properties (swelling degree, sorption capacity, and rheological and thermal properties). For instance, the CB hydrogels showed the largest hysteresis at values of RH > 50%, followed by the CE hydrogels, mainly due to the predominant shape of the pores, more precisely, the “ink bottle” shape for CB and the cylindrical shape for CE. The gel fraction was greater than 90% for the CE and CB hydrogels, suggesting the obtaining of crosslinked 3D structures that are stable in an aqueous medium at a relatively high temperature (60 °C). Regarding the swelling degree (Q_t_) for chemically crosslinked hydrogels, a clear influence of the type of crosslinking agent (mono-, bi-, and tri-epoxy) was observed. Thus, a decrease in Qt was recorded according to the series Qt CE > Qt CB > Qt CT, suggesting the formation of more compact networks as the number of epoxy groups in the structure of the crosslinker increased. These 3D crosslinked structures also led to exceptional rheological properties, especially regarding the elastic component of these hydrogels, where tan δ for the chemically crosslinked hydrogels < tan δ for the physically crosslinked hydrogel. Moreover, the high thermal stability of the CB hydrogel was remarkable with its ability to withstand temperatures close to 400 °C.

As a general conclusion, the results highlight the possibility of using specific crosslinking agents, such as zero-length ECH, dumbbell-shaped linear BDDE, or Y-shaped branched TMPTGE, in order to obtain hydrogels with tailored and guided characteristics to respond to certain requirements of a particular field of application. For example, if hydrogels with high a swelling degree and improved elasticity are required, such as materials for wound dressings, then it is preferable to use crosslinkers with reduced functionality (i.e., mono-epoxy) rather than with multi-epoxy groups. On the other hand, if more rigid hydrogels are necessary, with dense and compact matrices but with a lower swelling degree, then the choice must be directed towards multifunctional crosslinkers (bi-, tri-, or multi-epoxy) and, if possible, with long or branched alkyl chains.

## 4. Materials and Methods

### 4.1. Materials

Microcrystalline cellulose was purchased from Sigma-Aldrich (Saint Louis, MO, USA) under the trade name of Avicel PH-101 (~50 µm particle size; DP = 180). Epichlorohydrin (ECH, purity > 98%, M_w_ = 92.52 g/mol) was purchased from Merck (Hohenbrunn, Germany), 1,4-butanediol diglycidyl ether (BDDE, purity ≤ 100%, M_w_ = 202.25 g/mol) and trimethylolpropane triglycidyl ether (TMPTGE, purity > 95%, M_w_ = 302.36 g/mol) were purchased from Sigma-Aldrich (Saint Louis, MO, USA). All three crosslinkers were used without previous purification. Sodium hydroxide (NaOH) in pellets, with a purity ≥ 97%, was supplied by Merck (Hohenbrunn, Germany).

### 4.2. Methods and Equipment

#### 4.2.1. Hydrogels Preparation

The preparation of hydrogels was carried out by a two-step method, which was adapted from a previously reported method [10].

In the first stage, a suspension of 6% (*w*/*w*) cellulose in NaOH solution was achieved, which was subsequently frozen at −30 °C for 24 h, followed by a thawing stage at room temperature. Starting from this point, the physically crosslinked hydrogel (noted with C) was obtained by keeping the cellulose solution at room temperature for 24 h to undergo physical gelation, and then the obtained gel was carefully washed to coagulate cellulose and remove the NaOH. At the end, the hydrogel was lyophilized in a freeze dryer ALPHA 1-2/LD (Martin Christ Drying Systems GmbH, Osterode, Germany).

In the second stage, for the synthesis of chemically crosslinked hydrogels, 3.2 g of crosslinking agents (ECH, BDDE or TMPTGE) was added to the cellulose solution prepared in the first stage, under strong mechanical stirring for 10 min, until homogeneous solutions were obtained. Chemical crosslinking of the hydrogels was performed at a moderate temperature (40 °C) for 5 h. The obtained hydrogels (CE, CB, and CT) were washed with warm water (at 40 °C) for several days to remove the unreacted crosslinking agent or other impurities, and finally, they were lyophilized.

#### 4.2.2. Gel Fraction

The effectiveness of 3D network formation in hydrogels can be quantitatively evaluated based on gel fraction (GF, %). The stability of a regenerated hydrogel, due to its resistance to dissolution in hot water, is known as gelation degree. First, the hydrogels were oven dried at 40 °C until their weight was constant. The dried hydrogels were then immersed in a distilled water bath at 60 °C for 48 h. The insoluble part corresponding to the hydrogel was dried and weighed. All experiments were performed in triplicate and the data are expressed as the mean ± standard deviation (SD). The gel fraction was calculated using Equation (1) [27].

(1) $$\mathrm{GF}\;\left(\%\right)=\frac{M_{g}}{M_{0}}\times 100$$

where M_g_ is the weight of dried insoluble portion of hydrogel, after immersion(g) and M_0_ is the initial weight of the dry hydrogel, before immersion (g).

In order to estimate the amount of the crosslinker (AC, moles) consumed in the crosslinking reaction, we assumed that the cellulose (0.5 g) involved in the chemical crosslinking is found entirely in the mass of the final hydrogel, together with the crosslinking agent. Thus, the amount of crosslinker (AC, moles) consumed was calculated using Equation (2) and its further estimation was carried out referring to the number of moles of anhydroglucose units (m_AGU_, moles) resulting from Equation (3).

(2) $$\mathrm{AC}\;\left(\mathrm{moles}\right)=\frac{\left(M_{H}-M_{C}\right)}{M}$$

(3) $$m_{\mathrm{AGU}}\left(\mathrm{moles}\right)=\frac{M_{C}}{M_{\mathrm{AGU}}}=0.003086$$

where M_H_ is the weight of the final lyophilized hydrogels (g); M_C_ is the weight of cellulose used in the crosslinking process (0.5 g); M is the molar mass of crosslinker (g/mol); m_AGU_ is the number of moles of AGUs (moles); M_AGU_ is the molar mass of one anhydroglucose unit (162 g/mol).

#### 4.2.3. Swelling Degree and Swelling Kinetics

Initially, the maximum swelling degree (Q_max_, %) of the never-dried hydrogels, after washing for several days with warm distilled water, was determined using Equation (4).

(4) $$Q_{\max}\left(\%\right)=\frac{\left(M_{\mathrm{sw}}-M_{d}\right)}{M_{d}}\times 100$$

where M_sw_ is the weight of maximally swollen hydrogel (g); M_d_ is the weight of dry hydrogel through lyophilization (g). All experiments were performed in triplicate and the data are expressed as the mean ± SD.

Since the drying process is expected to influence the swelling process of the hydrogels, the swelling degree after lyophilization (Q_t_, %) was also determined using Equation (5).

(5) $$Q_{t}\left(\%\right)=\frac{\left(M_{t}-M_{d}\right)}{M_{d}}\times 100$$

where M_t_ is the weight of swollen hydrogel at time t (g); M_d_ is the weight of dry hydrogel (g). All experiments were performed in triplicate and the data are expressed as the mean ± SD.

At the same time, the study of swelling kinetics was also carried out in order to elucidate the way the water molecules diffuse into the hydrogel matrices over time. In this respect, the lyophilized samples were submerged in distilled water to swell, at 37 ± 1 °C. The samples were taken out of the water at predetermined time intervals, their surface was wiped with filter paper to remove the excess water, and their weights were recorded. The kinetics of the swelling process of the lyophilized hydrogels was carried out using Equation (6) in order to describe the Fickian or non-Fickian behavior of the swelling process [38].

(6) $$\frac{W_{t}}{W_{\mathrm{eq}}}={\;k}_{\mathrm{sw}}\times {\;t}^{n_{\mathrm{sw}}}$$

where W_t_ is the amount of water absorbed at time t by the hydrogel (g); W_eq_ is the amount of water absorbed by the hydrogel at equilibrium (g); k_sw_ is the swelling constant that incorporates the characteristics of the macromolecular network system (min^−1^); n_sw_ is the swelling diffusional exponent, which is indicative of the transport mechanism. The constants n_sw_ and k_sw_ were calculated from the slopes and intercepts of the plots of ln(W_t_/W_eq_) vs. ln(t). Usually, Equation (6) is applied in the early stages of the swelling process (Q_t_ < 60%), where the linearity is observed.

#### 4.2.4. Dynamic Vapor Sorption Measurements

The behavior of the hydrogels in atmosphere with controlled humidity was highlighted by dynamic vapor sorption (DVS) analysis, a gravimetric technique measuring, with an ultra-sensitive microbalance, the changes in sample weight in response to changes in relative humidity. The measurements were made in the test room at a constant temperature. DVS analysis was performed in the dynamic regime using fully automated IGAsorp gravimetric equipment manufactured by Hiden Analytical (Warrington, UK). The determinations were directed by a user-friendly software package, IGAsorp’s HIsorp software V6.50.42 (Hiden Analytical, Warrington, UK). The first step involved the placement of the sample in a special container, followed by its drying at 25 °C in flowing nitrogen (250 mL/min) until its weight reached equilibrium at a relative humidity (RH) less than 1% and, after that, followed the isotherm recording. The RH gradually increased from 0 to 90%, in 10% humidity steps, every step having a pre-established equilibrium time until the RH increased again. The amount of water absorbed by the hydrogels (W, %) was calculated using Equation (7). The data are also presented in terms of absolute hysteresis, which is the difference between the weight of adsorbed water under conditions of adsorption (W_ads_) and desorption (W_des_), obtained by subtracting W_ads_ from W_des_ at a given RH. Based on the water vapor sorption data, the specific surface area (m^2^/g) was also evaluated using the Brunauer–Emmett–Teller (BET) kinetic model (Equation (7)) [53], which allows for the modeling of sorption isotherms registered under dynamic conditions.

(7) $$W\;\left(\%\right)=\frac{W_{m}\mathrm{cRH}}{\left(1\;–\mathrm{RH}\right)\left(1\;–\mathrm{RH}\;+\mathrm{cRH}\right)}$$

where the parameters involved are W—the weight of adsorbed water (%); W_m_—the weight of water forming a monolayer (g); c—the sorption constant; RH—the relative humidity (%).

#### 4.2.5. Scanning Electron Microscopy Analysis

SEM was performed in order to thoroughly analyze the internal morphology of the hydrogels and to verify whether the crosslinking process impacted the pore structure. SEM is the most widely employed technique for the investigation of pore shape and pore size distribution of hydrogel networks. Before measurements, the cross-sections of the hydrogels were coated with a platinum layer (6 nm), using a Leica EM ACE200 Sputter coater (Leica Microsystem, Vienna, Austria). SEM analyses were performed on a Verios G4 UC Scanning Electron Microscope (Thermo Scientific, SEM, FEI Company, Brno, Czech Republic), using a secondary electron detector (Everhart–Thornley detector, ETD) with 10 kV accelerating voltage and a beam current of 0.8 nA. The average pore size and the standard deviation (SD) were determined by measuring 100 randomly chosen pores from the SEM micrographs exported into image analysis software (ImageJ software, v1.53k) [15].

#### 4.2.6. Attenuated Total Reflectance Fourier Transform Infrared Spectroscopy

Attenuated total reflectance Fourier transform infrared (ATR-FTIR) spectroscopy measurements were performed using a Bruker Vertex 70 spectrometer (Bruker Optics GMBh, Ettlingen, Germany), with a single-bounce diamond crystal at the incidence angle of 45°. Each spectrum consisted of an average of 128 scans at 2 cm^−1^ resolution in the 4000–600 cm^−1^ wavelength range.

#### 4.2.7. Rheology Measurements

The dynamic rheological behavior of the developed cellulose-based hydrogels was assessed by using an Anton Paar Physica MCR 301 rheometer (Graz, Austria), with plate-plate geometry (25 mm diameter, 4 mm gap), at a constant temperature (25 ± 0.1 °C). Before measurements, the hydrogel samples were swollen in distilled water at room temperature overnight, and the measurements were performed in triplicate at room temperature. A Peltier system for controlling temperature and evaporation was used during measurements. The amplitude sweep tests were performed within 0.001 and 100% strain range with a constant frequency of 1 Hz to determine the linear viscoelastic region (LVR—the region in which the modules are strain-independent) and to characterize the fracture process of the hydrogels. The viscoelastic properties of the hydrogels were also evaluated by performing the oscillatory frequency sweep tests, carried out within the determined LVR, in the frequency range from 0.05 to 500 s^−1^, at a constant strain of 1%.

#### 4.2.8. Differential Scanning Calorimetry and Thermogravimetric Analyses

Mettler Toledo DSC1 equipment (Columbus, OH, USA) was used to record the DSC curves. The tests were carried out for samples with a mass between 2.5 and 5.7 mg, with a heating rate of 10 °C/min, in an inert atmosphere (nitrogen). The scans were performed in a temperature range of 25–250 °C.

Thermogravimetric (TG) and derivative thermogravimetric (DTG) curves were recorded with Mettler Toledo 851^e^ equipment (Columbus, OH, USA). Sample mass varying between 2 and 5 mg was used. The measurements were done in nitrogen atmosphere, with a flow rate of 20 L/min and a heating rate of 10 °C/min. TG and DTG curves were recorded in the temperature range of 25–600 °C. After checking the reproducibility of the data obtained by recording three tests under the same conditions, it was found that the uncertainty was less than 1%.

The DSC, TG, and DTG curves were processed using STARe SW 9.10 software from Mettler Toledo in order to obtain the main thermal characteristics.

## Figures and Tables

**Table 1:** Schematic representation of the crosslinking agents’ structure and the crosslinked cellulose chains with the corresponding crosslinker: (a) epichlorohydrin (ECH, red color), (b) 1,4-butanediol diglycidyl ether (BDDE, green color), and (c) trimethylolpropane triglycidyl ether (TMPTGE, blue color) (ether bonds are highlighted).

(a)	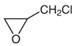	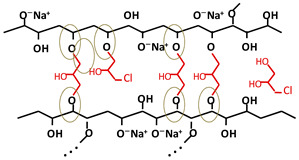
(b)	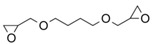	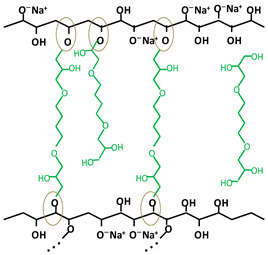
(c)	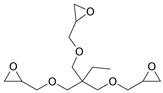	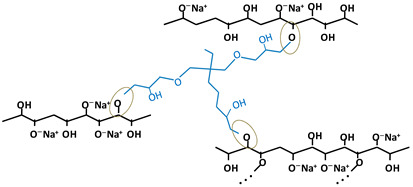

**Table 2:** Gel fraction and crosslinker consumption per unit of AGU, for the obtained hydrogels.

Sample	M_H_ (g)	M_H_/M_C_ Ratio	mole_AC_/mole_AGU_	GF (%)
C	0.37	0.74	-	96.3 ± 4.7
CE	0.75	1.50	0.876	97.3 ± 2.3
CB	1.65	3.30	1.843	94.5 ± 2.0
CT	1.36	2.72	0.915	82.3 ± 2.8

**Table 3:** The swelling degrees of never-dried (Q_max_) and dried (Q_t_) hydrogels, and kinetic parameters of the swelling process.

Sample	Q_max_ (%)	Q_t_ (%)	n_sw_	k_sw_	R^2^	Transport Mechanism
C	970 ± 87	710 ± 46	0.038	0.135	0.997	Pseudo Fickian diffusion
CE	1280 ± 54	1030 ± 23	0.094	0.246	0.992	
CB	940 ± 48	580 ± 23	0.111	0.358	0.996	
CT	970 ± 41	470 ± 18	0.104	0.516	0.996	

**Table 4:** The water content at maximum RH, and surface parameters evaluated based on adsorption/desorption isotherms.

Sample	W (%)	BET Data *	
		Surface Area (m^2^/g)	Monolayer (g/g)
C	22.02	237.6	0.0677
CE	36.61	266.4	0.0759
CB	28.97	374.9	0.1068
CT	15.82	114.3	0.0326

* BET data—determined based on the desorption branch of the isotherm (registered up to RH of 40%).

**Table 5:** Dynamic moduli (G′ = G″) and strain (γ) at the crossover point (from amplitude sweep tests), and values of G′, G″, and tan δ at 5 s^−1^ (from frequency sweep tests).

Sample	Amplitude Sweep Tests		Frequency Sweep Tests (at 5 s^−1^)		
	G′ = G″ (Pa)	γ (%)	G′ (Pa)	G″ (Pa)	tan δ
C	195,700	0.93	191,000	176,000	0.921
CE	10,920	45.1	38,100	3060	0.080
CB	540	68.4	30,700	4390	0.143
CT	3239	37.5	13,900	1400	0.101

**Table 6:** DSC data of cellulose-based hydrogels.

Sample	T_onset_ (°C)	T_peak_ (°C)	T_endset_ (°C)	ΔH (J/g)
C	36.3	84.6	178.2	148.5
CE	36.1	76.6	127.7	80.6
CB	31.2	70.6	118.2	51.0
CT	34.2	71.8	122.3	45.1

**Table 7:** Thermal degradation of cellulose-based hydrogels.

Sample	Stage I				Stage II				Total Residue (%)
	T_onset_ (°C)	T_peak_ (°C)	T_endset_ (°C)	Mass loss (%)	T_onset_ (°C)	T_peak_ (°C)	T_endset_ (°C)	Mass Loss (%)	
C	60.0	88.8	114.2	3.35	318	342	359	83.2	13.45
CE	55.6	67.5	93.3	1.84	332	372	407	86.2	11.96
CB	45.0	54.1	89.2	0.86	345	403	422	90.1	9.04
CT	47.9	62.0	99.1	0.50	322	388	416	89.0	10.50

## Data Availability

The original contributions presented in the study are included in the article, further inquiries can be directed to the corresponding author/s.

## References

[1] Udoetok I.A., Dimmick R.M., Wilson L.D., Headley J.V. (2016). Adsorption Properties of Cross-Linked Cellulose-Epichlorohydrin Polymers in Aqueous Solution. Carbohydr. Polym..

[2] Ciolacu F. (2018). Paper-Based Microfluidic Devices on Fibrous Platforms with Designed Structure. Cellulose Chem. Technol..

[3] Nasution H., Harahap H., Dalimunthe N.F., Ginting M.H.S., Jaafar M., Tan O.O.H., Aruan H.K., Herfananda A.L. (2022). Hydrogel and Effects of Crosslinking Agent on Cellulose-Based Hydrogels: A Review. Gels.

[4] Liang L., Bhagia S., Li M., Huang C., Ragauskas A.J. (2020). Cross-Linked Nanocellulosic Materials and Their Applications. ChemSusChem.

[5] Bonetti L., DeNardo L., Fare S. (2023). Crosslinking Strategies in Modulating Methylcellulose Hydrogel Properties. Soft Matter.

[6] Ursini O., Grieco M., Sappino C., Capodilupo A.L., Giannitelli S.M., Mauri E., Bucciarelli A., Coricciati C., deTurris V., Gigli G. (2023). Modulation of Methacrylated Hyaluronic Acid Hydrogels Enables Their Use as 3D Cultured Model. Gels.

[7] Mota L.O., Gimenez I.F. (2022). Cellulose-Based Materials Crosslinked with Epichlorohydrin: A Mini Review. Rev. Virtual Quim..

[8] Zyadeh M.T., Hamadneh I.M.K., Kasrawi M.A.R., Saadeh H., Shahein M.H. (2023). Synthesis of Cellulose-Based Hydrogel for Regulating the Release of Nitrogen Fertilizer. Cellul. Chem. Technol..

[9] Truong T.T.C., Bam V.V., Thi A.P.L., Phan N.T.T., Kobayashi T., Nga D.T.T., Nguyen K.D. (2024). Chemically Crosslinked Cellulose-Based Hydrogel Prepared from Rice Straw for the Removal of Aqueous Hexavalent Chromium Ion from Wastewater. Cellul. Chem. Technol..

[10] Ciolacu D.E., Rusu D., Darie-Nita R.N., Tîmpu D., Ciolacu F. (2022). Influence of Gel Stage from Cellulose Dissolution in NaOH-Water System on the Performances of Cellulose Allomorphs-Based Hydrogels. Gels.

[11] Guo H., Lei B., Yu J., Chen Y., Qian J. (2021). Immobilization of Lipase by Dialdehyde Cellulose Crosslinked Magnetic Nanoparticles. Int. J. Biol. Macromol..

[12] Liu H., Kar N., Edgar K.J. (2012). Direct Synthesis of Cellulose Adipate Derivatives Using Adipic Anhydride. Cellulose.

[13] Priya G., Narendrakumar U., Manjubala I. (2019). Thermal Behavior of Carboxymethyl Cellulose in the Presence of Polycarboxylic Acid Crosslinkers. J. Therm. Anal. Calorim..

[14] Korhonen O., Budtova T. (2019). Gelation of Cellulose-NaOH Solutions in the Presence of Cellulose Fibers. Carbohydr. Polym..

[15] Nicu R., Ciolacu D.E., Petrovici A.R., Rusu D., Avadanei M., Mihaila A.C., Butoi E., Ciolacu F. (2023). 3D Matrices for Enhanced Encapsulation and Controlled Release of Anti-Inflammatory Bioactive Compounds in Wound Healing. Int. J. Mol. Sci..

[16] Ciolacu D.E., Nicu R., Suflet D.M., Rusu D., Darie-Nita R.N., Simionescu N., Cazacu G., Ciolacu F. (2023). Multifunctional Hydrogels Based on Cellulose and Modified Lignin for Advanced Wounds Management. Pharmaceutics.

[17] Ahmad D.F.B.A., Wasli M.E., Tan C.S.Y., Musa Z., Chin S.F. (2023). Eco-friendly Cellulose-Based Hydrogels Derived from Wastepapers as a Controlled-Release Fertilizer. Chem. Biol. Technol. Agric..

[18] Martucci J.F., Espinosa J.P., Ruseckaite R.A. (2015). Physicochemical Properties of Films Based on Bovine Gelatin Cross-Linked with 1,4-Butanediol Diglycidyl Ether. Food Bioprocess Technol..

[19] DeBoulle K., Glogau R., Kono T., Nathan M., Tezel A., Roca-Martinez J.X., Paliwal S., Stroumpoulis D. (2013). A Review of the Metabolism of 1,4-Butanediol Diglycidyl Ether–Crosslinked Hyaluronic Acid Dermal Fillers. Dermatol. Surg..

[20] Chang L., Zhang J., Jiang X. (2019). Comparative Properties of Hyaluronic Acid Hydrogel Crosslinked with 1,4-Butanediol Diglycidyl Ether Assayed Using a Marine Hyaluronidase. IOP Conf. Ser. Mater. Sci. Eng..

[21] Wende F.J., Gohil S., Nord L.I., Kenne A.H., Sandström C. (2017). 1D NMR Methods for Determination of Degree of Cross-Linking and BDDE Substitution Positions in HA Hydrogels. Carbohydr. Polym..

[22] Tang S., Chi K., Xu H., Yong Q., Yang J., Catchmark J.M. (2021). A Covalently Cross-Linked Hyaluronic Acid/Bacterial Cellulose Composite Hydrogel for Potential Biological Applications. Carbohydr. Polym..

[23] Venzhik A.N., Nikolaev D.A., Romanova I.V. (2022). Study of Rheological and Structural Properties of Modified Carboxymethyl Cellulose Solutions Using Crosslinking Agents Based on Substituted Oxyranes. Inorg. Mater. Appl. Res..

[24] Su T., Wu L., Zuo G., Pan X., Shi M., Zhang C., Qi X., Dong W. (2020). Incorporation of Dumbbell-Shaped and Y-shaped Cross-linkers in Adjustable Pullulan/Polydopamine Hydrogels for Selective Adsorption of Cationic Dyes. Environ. Res..

[25] Yuan J., Du G., Yang H., Liu S., Wu Y., Ni K., Ran X., Gao W., Yang L., Li J. (2022). Functionalization of Cellulose with Amine Group and Cross-Linked with Branched Epoxy to Construct High-Performance Wood Adhesive. Int. J. Biol. Macromol..

[26] Ciolacu D., Rudaz C., Vasilescu M., Budtova T. (2016). Physically and Chemically Cross-Linked Cellulose Cryogels: Structure, Properties and Application for Controlled Release. Carbohydr. Polym..

[27] Salleh K.M., Zakaria S., Sajab M.S., Gan S., Chia C.H., Jaafar S.N.S., Amran U.A. (2018). Chemically Crosslinked Hydrogel and Its Driving Force towards Superabsorbent Behavior. Int. J. Biol. Macromol..

[28] Ruhr D., John M., Reiche A. (2021). Determination of the Effective Degree of Cross-Linking of Porous Cellulose Membranes Cross-Linked with Bifunctional Epoxides. Carbohydr. Polym..

[29] Lungu A., Cernencu A.I., Dinescu S., Balahura R., Mereuta P., Costache M., Syverud K., Stancu I.C., Iovu H. (2021). Nanocellulose-Enriched Hydrocolloid-Based Hydrogels Designed Using a Ca^2+^ Free Strategy Based on Citric Acid. Mater. Des..

[30] Amirjani A., Salehi K., Sadrnezhaad S.K. (2022). Simple SPR-Based Colorimetric Sensor to Differentiate Mg^2+^ and Ca^2+^ in Aqueous Solutions. Spectrochim. Acta Part A Mol. Biomol. Spectrosc..

[31] Ciolacu F., Ianus G., Marian G., Munteanu C., Paleu V., Nazar B., Istrate B., Gudîma A., Daraduda N. (2022). A Qualitative Assessment of the Specific Woody Biomass of Fruit Trees. Forests.

[32] Alzorqi I., Sudheer S., Lu T.-J., Manickam S. (2017). Ultrasonically Extracted β-D-Glucan from Artificially Cultivated Mushroom, Characteristic Properties and Antioxidant Activity. Ultrason. Sonochem..

[33] Luo H., Yin Y., Wang Y., Li Q., Tang A., Liu Y. (2022). Enhanced Properties of a Soybean Adhesive by Modification with a Cycloaliphatic Epoxy Resin. Int. J. Adhes. Adhes..

[34] Palacios Y.Y.L., Khandani S., Garcia E.P., Chen A., Wang S., Roy K., Knez D., Kim D.A., Rocha-Mendoza I., Potma E.O. (2024). Spectroscopic Analysis of the Sum-Frequency Response of the Carbon–Hydrogen Stretching Modes in Collagen Type I. J. Chem. Phys..

[35] Xu Y., Han Y., Chen M., Li J., Li J., Luo J., Gao Q. (2022). A Soy Protein-Based Film by Mixed Covalent Cross-Linking and Flexibilizing Networks. Ind. Crop. Prod..

[36] Luo Y., Li G., Chen L., Hong F.F. (2023). Preparation and Evaluation of Bacterial Nanocellulose/Hyaluronic Acid Composite Artificial Cornea for Application of Corneal Transplantation. Biomacromolecules.

[37] Zou P., Yao J., Cui Y.-N., Zhao T., Che J., Yang M., Li Z., Gao C. (2022). Advances in Cellulose-Based Hydrogels for Biomedical Engineering: A Review Summary. Gels.

[38] Bruschi M.L., Bruschi M.L. (2015). Mathematical Models of Drug Release. Strategies to Modify the Drug Release from Pharmaceutical Systems.

[39] Nistor A., Stiubianu G., Racles C., Cazacu M. (2011). Evaluation of the Water Sorption Capacity of Some Polymeric Materials by Dynamic Vapour Sorption. Mater. Plast..

[40] Thommes M., Kaneko K., Neimark A.V., Olivier J.P., Rodriguez-Reinoso F., Rouquerol J., Sing K.S.W. (2015). Physisorption of Gases, with Special Reference to the Evaluation of Surface Area and Pore Size Distribution. Pure Appl. Chem..

[41] Uimonen T., Hautamäki S., Altgen M., Kymäläinen M., Rautkari L. (2020). Dynamic Vapour Sorption Protocols for the Quantification of Accessible Hydroxyl Groups in Wood. Holzforschung.

[42] Morishige K. (2021). Revisiting the Nature of Adsorption and Desorption Branches: Temperature Dependence of Adsorption Hysteresis in Ordered Mesoporous Silica. ACS Omega.

[43] Kachrimanis K., Noisternig M.F., Griesser U.J., Malamataris S. (2006). Dynamic Moisture Sorption and Desorption of Standard and Silicified Microcrystalline Cellulose. Eur. J. Pharm. Biopharm..

[44] Szymaszek P., Tomal W., Świergosz T., Kamińska-Borek I., Popielarz R., Ortyl J. (2023). Review of Quantitative and Qualitative Methods for Monitoring Photopolymerization Reactions. Polym.Chem..

[45] Seera S.D.K., Kundu D., Banerjee T. (2020). Physical and Chemical Crosslinked Microcrystalline Cellulose-Polyvinyl Alcohol Hydrogel: Freeze–Thaw Mediated Synthesis, Characterization and In Vitro Delivery of 5-Fluorouracil. Cellulose.

[46] Xu J., Boddu V.M., Liu S.X. (2022). Rheological Properties of Hydrogels Produced by Cellulose Derivatives Crosslinked with Citric Acid, Succinic Acid and Sebacic Acid. Cellul. Chem. Technol..

[47] Young A.T., White O.C., Daniele M.A. (2020). Rheological Properties of Coordinated Physical Gelation and Chemical Crosslinking in Gelatin Methacryloyl (GelMA) Hydrogels. Macromol. Biosci..

[48] Blažic R., Marušic K., Vidovic E. (2023). Swelling and Viscoelastic Properties of Cellulose-Based Hydrogels Prepared by Free Radical Polymerization of Dimethylaminoethyl Methacrylate in Cellulose Solution. Gels.

[49] Jiang X., Yang X., Yang B., Zhang L., Lu A. (2021). Highly Self-Healable and Injectable Cellulose Hydrogels via Rapid Hydrazone Linkage for Drug Delivery and 3D Cell Culture. Carbohydr. Polym..

[50] Bhattacharya S., Shunmugam R. (2020). Unraveling the Effect of PEG Chain Length on the Physical Properties and Toxicant Removal Capacities of Cross-Linked Network Synthesized by Thiol–Norbornene Photoclick Chemistry. ACS Omega.

[51] Kono H. (2014). Characterization and Properties of Carboxymethyl Cellulose Hydrogels Crosslinked by Polyethylene Glycol. Carbohydr. Polym..

[52] Cortés-Triviño E., Valencia C., Delgado M.A., Franco J.M. (2018). Rheology of Epoxidized Cellulose Pulp Gel-like Dispersions in Castor Oil: Influence of Epoxidation Degree and the Epoxide Chemical Structure. Carbohydr. Polym..

[53] Ciolacu D., Doroftei F., Cazacu G., Cazacu M. (2013). Morphological and Surface Aspects of Cellulose-Lignin Hydrogels. Cellulose Chem. Technol..

